# Controlling Clinical States Governed by Different Temporal Dynamics With Closed-Loop Deep Brain Stimulation: A Principled Framework

**DOI:** 10.3389/fnins.2021.734186

**Published:** 2021-11-11

**Authors:** Gerd Tinkhauser, Eduardo Martin Moraud

**Affiliations:** ^1^Department of Neurology, Bern University Hospital and University of Bern, Bern, Switzerland; ^2^Department of Clinical Neurosciences, Lausanne University Hospital, Lausanne, Switzerland; ^3^Defitech Center for Interventional Neurotherapies (.NeuroRestore), Ecole Polytechnique Fédérale de Lausanne and Lausanne University Hospital, Lausanne, Switzerland

**Keywords:** closed-loop DBS, local field potentials (LFP), basal ganglia, Parkinson’s disease, multi-objective control

## Abstract

Closed-loop strategies for deep brain stimulation (DBS) are paving the way for improving the efficacy of existing neuromodulation therapies across neurological disorders. Unlike continuous DBS, closed-loop DBS approaches (cl-DBS) optimize the delivery of stimulation in the temporal domain. However, clinical and neurophysiological manifestations exhibit highly diverse temporal properties and evolve over multiple time-constants. Moreover, throughout the day, patients are engaged in different activities such as walking, talking, or sleeping that may require specific therapeutic adjustments. This broad range of temporal properties, along with inter-dependencies affecting parallel manifestations, need to be integrated in the development of therapies to achieve a sustained, optimized control of multiple symptoms over time. This requires an extended view on future cl-DBS design. Here we propose a conceptual framework to guide the development of multi-objective therapies embedding parallel control loops. Its modular organization allows to optimize the personalization of cl-DBS therapies to heterogeneous patient profiles. We provide an overview of clinical states and symptoms, as well as putative electrophysiological biomarkers that may be integrated within this structure. This integrative framework may guide future developments and become an integral part of next-generation precision medicine instruments.

## Closed-Loop Deep Brain Stimulation: Toward Multi-Objective Control Algorithms in Space and Time

Deep brain stimulation is an established treatment option for patients with movement disorders [Parkinson’s disease (PD), Essential Tremor, and Dystonia], as demonstrated in randomized controlled trials (Krack et al., 2019). Current therapies are based on a constant delivery of stimulation with fixed parameters. Amplitude and contact selection are manually adjusted by clinicians, and then usually remain unchanged until follow-up clinical visits. Albeit widely spread and highly efficacious to alleviate predominant symptomatic traits, the static nature of this “one fits all the time” approach cannot account for all symptom fluctuations or manifestations that are episodic in nature (Lozano et al., 2019).

Closed-loop strategies offer the possibility to optimize DBS by automatically adjusting the timing and parameters of stimulation in real time based on biomarkers (Bronte-Stewart et al., 2020). The adaptability of these approaches helps ensure a maximal clinical benefit, sustained over time, while minimizing side-effects. In this loop, sensing (feedback) and stimulation (actuation) components need to be tuned to match the dynamical properties of the targeted manifestation. A broad variety of stimulation strategies and biomarkers have flourished over the past years to address the limitations of constant stimulation, for instance by specifically controlling ON-OFF fluctuations, reducing side-effects, and additionally to give answer to symptoms that are not optimally addressed by standard protocols, such as freezing of gait.

A putative limitation of current closed-loop strategies is their restricted scope, in which biomarkers, controller design and parameter choices are optimized to a unique symptom or neural manifestation in isolation. However, clinical states are dynamic, multi-faceted, and inter-connected. Some operate at the millisecond range while others evolve over many hours. They can occur independently or influence each other. Consequently, even though aforementioned closed-loop approaches showed improved efficacy over standard continuous DBS in well-controlled research conditions, the question of whether a satisfactory 24 h therapeutic coverage of multiple symptoms may be achieved with such strategies is far from clear.

A global framework is critically missing to guide the integration of all these developments into a clinically relevant therapeutic portfolio that exploits recent advances in implantable neurotechnologies (Cagnan et al., 2019; Gunduz et al., 2019; Parastarfeizabadi et al., 2020). This integrative framework needs to be modular, flexible, and easily adaptable by clinicians. It also needs to offer the possibility to address multiple symptoms while robustly dealing with dependencies that exist between clinical states, or interferences between parallel therapies.

We suggest that the structure of clinical and neurophysiological manifestations, segregated in time and space over multiple layers (see section below), may be mirrored by control strategies to steer the design of modular therapies embedding parallel control loops. This principled framework may guide future developments and become an integral part of next generation closed loop DBS systems.

## Myriad Temporal Scales of Clinical and Neurophysiological Manifestations

### Clinical Manifestations

Motor and non-motor symptoms exhibit highly diverse temporal properties. They emerge at different timepoints, progress at various speeds over the course of the disease, and diurnally fluctuate in intensity with according to their own variable time-constants. These distinct temporal behaviors are further intertwined since clinical manifestations can occur simultaneously or influence each other, adding a layer of complexity in the management of symptoms. For instance, tremor oscillations (∼5 Hz, 5 oscillations per second) stand in contrast to slow-changing states such as a dopaminergic wearing off episodes, which affect the condition of patients in the range of hours. Yet both states can be temporally related, as the likelihood of tremor episodes in PD may increase during wearing OFF dopaminergic states. Moreover, throughout the day, patients are engaged in different physiological states such as walking, talking, or sleeping, which may also continuously or intermittently be affect by disease-specific symptoms.

### Neurophysiological Manifestations

Signals to control DBS may be derived from neural recordings in the brain, peripheral sensors, or a combination of sources. Neural signals may encode various slow- or fast-changing states. Even depending on the way they are analytically processed, a same biomarker may be used to regulate control loops operating at different time scales. For instance, the better explored closed-loop DBS approaches for PD have employed beta oscillations in subthalamic nucleus (STN), which correlate with bradykinesia and rigidity (Brown et al., 2001; Neumann et al., 2016). Closed-loop approaches targeted either fast transient states of excessive synchrony (in the range of milliseconds) (Little et al., 2013; Moraud et al., 2018; Velisar et al., 2019) or instead beta activity fluctuations in the range of minutes to hours. These examples highlight the capacity to exploit the same biological signal *via* different temporal dynamics to address the same or various clinical goals.

A comprehensive understanding of the temporal properties governing different clinical manifestations and neurophysiological signatures, along with their interdependencies, is thus critical for the design of therapies that can optimally address multiple states in parallel. We outline a selection of different clinical and neurophysiological layers relevant to closed-loop therapies.

## Tremor

Across disorders, tremor tends to appear episodically (lasting from less than minutes up to hours), favored for instance by insufficient pharmacological control or agitation (Louis and Machado, 2015). Tremor occurrence is also influenced by motor states, as for example in PD tremor occurs prominently during rest, while in ET tremor is more pronounced during actions (Thenganatt and Jankovic, 2016).

Biomarkers and closed-loop strategies: Approaches for closed-loop DBS explored multiple control sources and control paradigms. Some used peripheral sensors to measure the amplitude of movements in the tremor frequency range (Yamamoto et al., 2013; Malekmohammadi et al., 2016) or delivered burst of stimulation locked to specific tremor phases (Cagnan et al., 2013, 2014, 2017). Tremor could also be detected from brain signals, either indexed by the lower frequency components (3–7 Hz) or more accurately by using machine-learning techniques allowing to combine multiple features from the whole-spectrum LFP (Hirschmann et al., 2017; Shah et al., 2018). Additionally, the action-induced occurrence of tremor in ET leveraged the development of closed-loop DBS algorithms with voluntary movement related modulations in LFPs as triggers for stimulation (Herron et al., 2017; Houston et al., 2017; Tan et al., 2018; He et al., 2020, 2021). All approaches ended up being tuned to operate in the range of milliseconds to multiple seconds.

## Gait and Gait Disturbances

Gait and balance deficits are common in PD, and induce a broad range of impairments including reduced arm swing and step length, shuffling steps, festination, freezing of gait or lack of postural control (Fasano et al., 2015). This phenomenological and temporal diversity, which include both continuous and episodic manifestations that are often interconnected, are difficult to treat. The effect of DBS on gait deficits is variable and patient specific spanning, from improvement to even worsening of gait (Hausdorff et al., 2009; Pötter-Nerger and Volkmann, 2013; Barbe et al., 2020).

Biomarkers and closed-loop strategies: During gait execution, alternating right and left gait cycles (1–2 Hz) are accompanied by periodic, time-locked modulations in the beta and gamma band power in STN LFP (Fischer et al., 2018; Hell et al., 2018). Recent work showed that alternating right and left DBS patterns, delivered intermittently at similar frequencies, could entrain stepping movements and increase gait regularity (Fischer et al., 2020; Wang and Choi, 2020). Additionally, beta modulations exhibit a degree of spectral segregation, with a stronger modulation in the high-beta range during leg vs. arm movements, (Fischer et al., 2018; Tinkhauser et al., 2019), which helped discriminate walking vs. standing (Canessa et al., 2020).

In addition, how freezing of gait (FoG) episodes could be delineated and targeted remains unanswered. In contrast to the alternating neuronal activity patterns during locomotion, FoG has been linked to the occurrence of prolonged bursts of beta activity (Anidi et al., 2018) and first data show promising results for beta-triggered cl-DBS to prevent FoG (Petrucci et al., 2020). Interestingly, the increase in beta activity associated with freezing of gait is more evident in the lower beta frequency ranges (15–21 Hz) and is also accompanied by an increase in the theta (5–8 Hz) activity (Chen C.-C. et al., 2019). Moreover, the electrophysiological signatures for vulnerability of freezing may be maintained >5 s and shows some degree of spatial segregation, as the theta power increase is more evident in the ventral part of the STN and in the substantia nigra. In line with this observation, stimulation at lower frequencies, or through ventral electrodes, has been suggested as option to reduce the occurrence of FoG (Sidiropoulos et al., 2013; Valldeoriola, 2019).

Considering these multi-faceted manifestations, therapies may need to flexibly combine (i) continuous adaptations in DBS during gait execution, as well as (ii) actively switching settings to improve and stabilize locomotion and prevent FoG (Fischer et al., 2020; Wang and Choi, 2020).

## Speech

Progressive speech impairments are common in various neurological disorders. Both in PD and ET, DBS often leads to further deterioration of speech performance which plays a limiting factor in the optimization of DBS (Hariz et al., 2008).

Biomarkers and closed-loop strategies: Closed-loop DBS may prevent speech deterioration, which is often encountered as a side-product during continuous DBS (Little et al., 2016b). It may do so by reducing the overall current spread to capsular structures, as an indirect effect of closed-loop DBS targeting other clinical manifestations (Little et al., 2016a,b). Speech could also actively be integrated in stimulation control loops, for instance by recognizing speech from brain signals or peripheral sensors. Recent data suggest that the STN is involved in speech processing, with articulator-specific information being spatially and temporally organized within the target structure (Chrabaszcz et al., 2019). In addition, and currently more easily, speech could be recognized from peripheral sensors, that might also allow to extract information of the clinical state and to help calibrate stimulation parameters (Rusz et al., 2015; Akçay and Oğuz, 2020).

## Symptom Fluctuations in Parkinson’s Disease

The later stages of PD are characterized by fluctuations of motor and non-motor symptoms that are difficult to control with standard therapies (Martínez-Fernández et al., 2016). ON/OFF fluctuations evolve in the range of minutes to hours, with transitions that become faster, more abrupt and less predictable as the disease progresses.

Biomarkers and closed-loop strategies: Currently, beta activity recorded from the basal ganglia (particularly the STN), represents the best-characterized biomarker to inform about drug-induced fluctuations, bradykinesia and rigidity (Brown et al., 2001; Kuhn et al., 2006; Hammond et al., 2007; Tinkhauser et al., 2017b). Different temporal scales may be considered to interact and influence beta activity.

### Fast Beta Modulations

Physiologically beta activity appears as short bursts (100 and 200 ms) (Feingold et al., 2015). However, in untreated PD patients, beta bursts are prolonged (between 200 and 1,000 ms) with higher amplitudes, both of which correlate with the level of clinical impairment (bradykinesia and rigidity) (Tinkhauser et al., 2017a,b, 2018; Duchet et al., 2021a). The direct impact of such temporally refined bursting dynamics on motor performance has been confirmed (Torrecillos et al., 2018; Khawaldeh et al., 2020, 2021; Tinkhauser et al., 2020). Therapies need to operate with a temporal resolution that matches the millisecond range, in order to properly detect and react to such fast-changing dynamics. One clinically successful approach computes beta power over a moving average of 400 ms (Little et al., 2013) and triggers stimulation whenever the windowed beta activity would surpass a pre-defined threshold, which allows to selectively trim pathologically long bursts (Tinkhauser et al., 2017a; Moraud et al., 2018). Another study processed the beta envelope using a larger timescale (800 ms) (Velisar et al., 2019), which might be at the limit to depict bursts. Importantly, during exposure to dopaminergic medication (Kuhn et al., 2006), beta bursts become shorter in duration and smaller in amplitude, hence they become more alike physiological bursts (Tinkhauser et al., 2017b). Closed-loop algorithms that track beta bursts would allow to take medication-induced changes into account to avoid cumulative (drug + stim) effects (Little et al., 2016a).

### Slow Beta Modulations

The temporal dynamics of beta activity can also be processed at longer temporal scales, with time-constants in the range of minutes. This processing does not capture beta burst dynamics, but instead accounts for clinical OFF/ON fluctuations related to medication intake. Adaptive DBS trials using this temporal resolution have been successfully piloted with a smoothing time constant of 50 s and a slow proportional controller that adapted stimulation accordingly (Rosa et al., 2017; Arlotti et al., 2018). A direct comparative study has demonstrated superiority of this closed-loop DBS approach over continuous DBS in improving motor UPDRS and reducing dyskinesias (Bocci et al., 2021).

### Finely Tuned Gamma

60–90 Hz frequency activity detected in the electrocorticogram, has been linked to the presence of dyskinesia in the ON medication state, and represents a promising electrophysiological biomarker to regulate DBS for such manifestations (Swann et al., 2016). This approach has been tested with a time constant of 30 s on narrow band gamma activity followed by a 600 ms decision window for stimulation control (Swann et al., 2018). However, the full electrophysiological and clinical picture of finely tuned gamma activity (FTG) still needs to be characterized, as stimulation-induced FTG measured in the STN and coherent to cortical activity, can also occur OFF medication and in the absence of dyskinesia (Wiest et al., 2021). Similarly the FTG frequency peak seems to differ in the OFF and ON medication state (Swann et al., 2018; Muthuraman et al., 2020; Wiest et al., 2021). Interestingly, the occurrence and duration of FTG can outlast stimulation delivery by (on average) 20 s, or even appear for the first time after stimulation (Wiest et al., 2021). The first chronic recordings during varying medication and stimulation states are now available and will help to refine the properties and value of FTG as well as other biomarkers (Gilron et al., 2021).

Current knowledge already delineates how control algorithms may need to follow and integrate different temporal dynamics of distinct biomarkers.

## Circadian Rhythmicity

Standard DBS therapies assume that the patient is in the same clinical state throughout the whole 24-h cycle. However, PD can be associated with different sleep problems such as REM-sleep behavior disorders (RBD), which can range from seconds to minutes, and alterations of sleep architecture. Several studies reported that STN DBS has a deepening and consolidating impact on nocturnal sleep (Baumann-Vogel et al., 2017; Zuzuárregui and Ostrem, 2020).

Biomarkers and closed-loop strategies: It is not yet clear how DBS should optimally act during sleep. Treatment goals and stimulation parametrization are likely to be different than those during daytime. Sleep therapies could potentially be optimized by considering sleep architecture and pathological sleep phenomena. An important prerequisite is the recognition of sleep stages, so that therapies may adapt to their specific requirements. NREM 1–3 and REM stages alternate cyclically, as defined by standard 30-s epochs classification systems (AASM, 2020). Recordings from the STN during sleep show similar sleep related oscillatory patterns as during polysomnography surface EEG (Urrestarazu et al., 2009; Thompson et al., 2018). Sleep stage information may be derived in real time with a high prediction accuracy of 91% (Christensen et al., 2019). In this latter work, the time-evolving spectra had a 15 s time constant and 0.5 Hz frequency resolution, which was sufficient to detect transitions. Shorter time-constants have also been proposed (Chen Y. et al., 2019). Multi-layered closed-loop control that differently reacts during wakefulness and sleep are becoming necessary, as supported by the observation that STN beta activity is high during REM sleep (similar as during wakefulness), but decreases with deeper sleep stages (N1->N3) (Urrestarazu et al., 2009). Hence, a closed-loop algorithm solely based on the daily beta profile, is likely to decrease stimulation toward NREM 3 and increase during REM sleep.

## Leveraging Temporal Dynamics to Enable Multi-Objective Closed-Loop DBS

**FIGURE 1:**
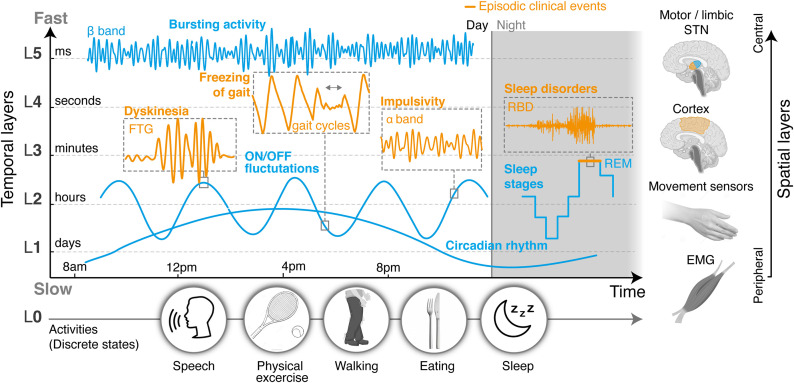
Layered organization of control sources, segregated in time and space. Clinical and neural manifestations, both episodic and non-episodic, evolve according to distinct time-constants that can be categorized into a range of temporal layers, from milliseconds (fastest layer, L5) to days (slowest layer, L1). An additional layer (L0) could capture changes in manifestations related to discrete states, such as daily activities, and operate in parallel to the others. In this proposed modular structure, layers may not be independent, since manifestations at different timescales can affect each other. Thus cross-layer interactions must also be accounted for. On top of differences in their temporal properties, neural processes may also be spatially segregated, and picked up from different locations in the brain or the periphery. Hence temporal and spatial layers may be combined to simplify, cluster, or distribute how symptoms are optimally monitored, detected, and addressed. Overall, this representation establishes a conceptual framework by which the clinical state of a patient can be described as the modular superposition of parallel, yet inter-dependent manifestations segregated in time and space. Closed-loop control approaches may mirror this layered organization in the design of multi-objective therapies that can concurrently address multiple symptoms, while suitably dealing with dependencies. L, temporal layer; STN, subthalamic nucleus; ON/OFF, with/without medication; FTG, finely tuned gamma oscillations.

Despite the heterogeneity of clinical manifestations and neurophysiological signatures, the time-constants that govern their individual behaviors may be categorized into discrete temporal layers (Figure 1). This layered organization makes it possible to simplify, cluster or distribute how multiple manifestations are jointly monitored and addressed. For instance, manifestations evolving in the millisecond range require sensing and control loops to operate at fast time scales, using algorithms that are computationally efficient and simple in complexity (e.g., PID or bang-bang control). Slower manifestations may instead use model-based control approaches that additionally include predictions in the control loops. Accounting for dependencies between manifestations as well as interfaces between controllers may be feasible.

Cross-layer interferences inevitably arise in multi-objective control. They happen when one control loop (for instance, regulating manifestation 1) induces (directly, or indirectly) a change in manifestation 2, which in turn triggers a response in a second control loop, and so on. If unaccounted for, interferences may lead controllers to diverge. Importantly, interferences are less likely to occur when controlled variables have different time constants. This “temporal decoupling” allows to pause one therapy, for instance a slow controller operating in the hour range, and to temporarily deliver another one (a fast controller reacting to an episodic event in the range of seconds), without much impact on the earlier.

**FIGURE 2:**
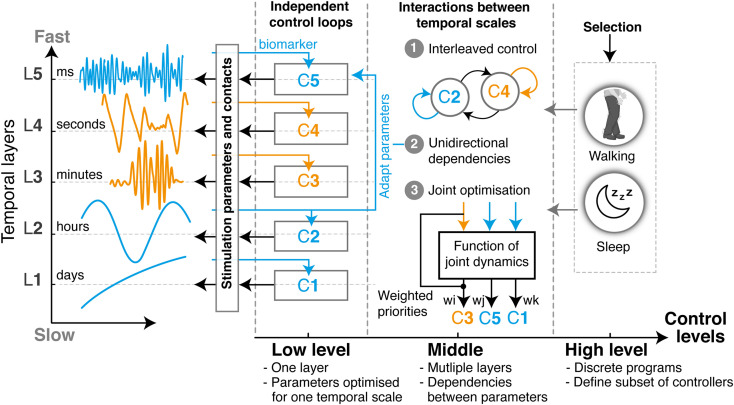
Layered multi-objective control for closed-loop DBS. Multi-objective control approaches for closed-loop DBS may mirror the layered structure of clinical and neural manifestations to optimize the control of multiple symptoms occurring concurrently or intermittently. Such a structure enables to distribute complexity over three incremental levels: The low level is composed of parallel independent control loops, each one operating at a unique temporal layer. The design and tuning of each controller (indicated as C1–C5) can thus be optimized for the specificities of a single manifestation, independently from changes that may arise from dependencies with other layers. Examples of closed-loop DBS in the literature, such as beta burst-based control of pathological beta synchrony, or gamma-driven control of dyskinesias, have been predominantly restricted to this layer. Second, cross-layer dependencies and interactions can be implemented in the middle level to address multiple symptoms. These may use (1) state machine controllers for switching between low-level controllers, (2) unilateral dependencies that tune the parameters of one layer using feedback from another, or (3) optimization controllers able to jointly tune various low-level control parameters based on a weighted evaluation of various manifestations, side effects and interferences. Finally, the high-level control layer enables the automatic selection of programs, which fit the discrete states occurring in layer L0. Each program will activate (or de-activate) a subset of low- and middle-level controllers, while specifying sensing channels and stimulation parameters. For instance, the way controllers might need to respond during sleep may not be the same as during the day. Overall, with this organization, multi-objective control therapies can easily be built, tuned, and combined in a modular way for each individual patient. All existing algorithmic approaches for closed-loop control proposed over the past years would easily be integrated under such structure.

Overall, the complexity of developing therapies that can address multiple manifestations may be distributed over three hierarchical levels of operation (Figure 2): the lowest level embeds closed-loop control algorithms that are optimized for individual manifestations, each one operating at a single temporal layer, regardless of other parallel ones. For instance, one controller may monitor beta band modulations and trim pathological beta bursts in the millisecond range, while another may track gamma band activity and identify dyskinetic episodes in the second to minute range. Most existing closed-loop DBS strategies developed to date could be integrated within this level. Second, a middle level manages the combined outcomes of low level-controllers and accounts for cross-layer dependencies and interferences that arise when two or more therapies operate concurrently. This control level ensures that multiples objectives are being respected. Finally, a higher-level encodes discrete programs or activities, which activate (or de-activate) subsets of low- and middle-level control loops. This higher level may either be automatically or manually selected by patients or clinicians, for instance to switch between day or night modes, or for specific tasks.

We propose that this hierarchical organization may simplify the design of multi-objective control therapies, while allowing to easily integrate existing algorithmic strategies for closed-loop DBS. In this integrative framework, control loops targeting different temporal layers are combined in a modular manner and operate in parallel (Figure 2). For a given patient, specific modules may easily be activated, and their joint operations managed to establish suitable therapeutic strategies that target all required manifestations.

### Low-Level Controllers: Targeting Individual Manifestations

A variety of control strategies have been proposed for addressing individual manifestations through closed-loop DBS. They relied predominantly on fast control approaches, either bang-bang controllers triggered by one (Little et al., 2013; Pina-Fuentes et al., 2019) of two (Velisar et al., 2019) thresholds, or using PID controllers (Rosa et al., 2017). These strategies relied predominantly on neural feedback from local field potentials (beta power from the STN (Little et al., 2013), gamma or theta power from cortical signals (Swann et al., 2018; Johnson et al., 2021) or movement measures (Cagnan et al., 2017). Few feedforward components that use predictive models have been included in real-life applications, even though biophysical or data-driven black-box models may greatly improve accuracy (Gorzelic et al., 2013; Su et al., 2019), especially for slowly changing biomarkers. To date, modeling has predominantly been used to better understand the dynamics of manifestations (Holgado et al., 2010; Fleming et al., 2020a), the impact that DBS may have on the circuits (Hahn and McIntyre, 2010; Weerasinghe et al., 2019; Fleming et al., 2020b) and to suggest possible control strategies for closed-loop DBS (Holt et al., 2016; Duchet et al., 2021b).

Examples from other neural engineering applications highlight the benefits of data-driven predictive models in closed-loop therapies. These used either movement sensor data or neural signals (commonly intra-cortical signals, with >100 channels) to control prostheses or robotic systems (Ethier et al., 2012; Hochberg et al., 2012), spinal cord stimulation for restoring movement (Wenger et al., 2014; Moraud et al., 2016; Bonizzato et al., 2018) and hemodynamic instability (Squair et al., 2021), or peripheral nerve stimulation for sensory feedback (Raspopovic et al., 2014). Many of these approaches may be easily integrated as low-level control loops within the proposed framework.

### Middle Control Level: Managing Multiple Objectives

**FIGURE 3:**
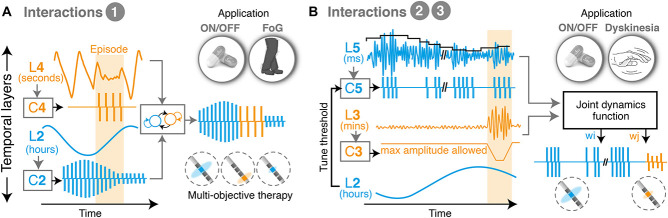
Illustrative examples of multi-objective therapies. **(A)** Example of state machine controller, which transitions between two different low-level control loops and interleaves two therapies for symptoms overlapping in time. This approach is suitable to combine clinical manifestations that exhibit markedly different temporal time-constants, and which consequently suffer from minimal crosstalk. For instance, episodic events such as freezing of gait, which happen in the range of seconds to minutes, may be easily interleaved with therapies that control ON-OFF fluctuations, which operate in the range of hours. In this configuration, each controller independently monitors and responds to a biomarker, and delivers an output optimized for its individual manifestation. The middle level controller activates one or the other, depending on the requirements over time. **(B)** Example of multi-objective therapy addressing manifestations on three temporal layers (namely, beta burst activity patterns, ON-OFF fluctuations and dyskinesias). Unidirectional dependencies are used to tune the controller of one layer based on feedback from another, in this case to set a threshold for detecting beta bursts (at the millisecond range) while accounting for fluctuations in beta power over the course of the day. In parallel, a joint optimization controller computes optimal stimulation amplitudes to minimize episodic stimulation-induced dyskinesias, based on a weighted evaluation of the need to trim pathologically long beta bursts while preventing side-effects. L, temporal layer; C, controller; w, weight; ON/OFF, with/without medication; FoG, freezing of gait.

The variety of clinical and neurophysiological manifestations also shapes the choice of middle-level control strategies to manage the joint outcome of multiple objectives, and critically affects the robustness and stability when addressing them concurrently. We outline various approaches that may be used:

1. State machine controllers allow to switch between independent states and make it possible to deliver various therapies in an interleaved manner. This approach is simple and easy to tune, as it only requires a few parameters (transitions). However, state machines do not directly manage interferences. They are thus most useful for processes that operate at clearly distinct layers (Toth et al., 2020). Examples in the literature using such approaches have been proposed for addressing beta bursts and episodic events such as FoG (Bronte-Stewart et al., 2020; Figure 3).
2. Unidirectional adaptations allow to tune the parameters of one controller using feedback of another temporal layer. This cross-layer interaction makes it possible to link two (or more) temporal layers, regardless of how far apart layers are. Examples include tuning the threshold for detecting beta-bursts based on feedback of ON-OFF fluctuations (Figure 3).
3. Optimization controllers employ a function of joint dynamics and are thus able to intrinsically account for interactions between manifestations. They provide the best way to deal with dependencies and are appropriate for layers that have similar time-constants (i.e., manifestations operating at similar dynamics and overlapping). However, these strategies are complex and require building models of the underlying processes and their responses to stimulation. Models may use biophysical, population-based or data-driven black box (machine learning) approaches, and allow to include feedforward and feedback control loops to more accurately control multiple objectives (Neumann and Rodriguez-Oroz, 2021).

Overall for a specific patient, the implementation of a suitable therapy capable of addressing multiple symptomatic traits would involve (i) establishing the patient profile, his/her specific therapeutic requirements and their time constants (similar to a “system identification” step), (ii) establishing what low-level controllers (modules) need to be activated to address each one of these manifestations, (iii) defining the neuro-physiological signatures or feedback signals that will drive each low-level controller, along with the objective to be achieved by each one of them, (iv) calibrating the individual parameters of each controller, e.g., threshold values, control coefficients, adaptation rates (optimized for each module in isolation), (v) defining possible inter-dependencies and interferences, and the best way to address them (choice of middle-level control type) based on the number of low-level controllers, their expected crosstalk and the relative importance of the manifestations that they monitor (defining priorities), (vi) evaluate the stability of the combined control strategy and establishing boundaries and safety measures to prevent divergence. This process may be repeated separately for each discrete program (high level control layer) e.g., one for the day and one for the night.

Steps (i–vi) may need to be done iteratively, over multiple sessions. Modularity would allow to incrementally refine therapies, adding one low-level controller at a time and tuning middle-layer control strategies accordingly to cope with added modules.

To increase stability and robustness over time, each low-level controller may include a self-adaptation term that tracks changes occurring over time and slowly adapts (Zaknich, 2005). Its implementation will strongly depend on the control type and the temporal layer on which it operates: Controllers with slow time-constants may exploit daily periodicity to track changes occurring from day to day, and use a forgetting factor that iteratively updates control parameters (e.g., daily update based on an average biomarker value over the previous day). Controllers that regulate episodic events such as freezing of dyskinesia may iteratively update control parameters after every few episodes.

## Technological Implications

Beside the conceptual framework of multi layered control, the technological requirements (hardware and software) to implement such comprehensive closed-loop strategies should not be left unmentioned. There are crucial technical capability demands for neurostimulators in the future. For instance, devices need to be able to monitor and differentially process multiple electrophysiological brain biomarkers and integrate them in the decision-making process (as outlined above). Such co-processing capabilities to flexible handle multiple inputs have been piloted (Stanslaski et al., 2018), and need to be refined in the future. As aforementioned, brain biomarkers can come from multiple sources (cortex, basal ganglia) including their corresponding somatotopic subdivisions, thus neurostimulators must be capable to handle multiple independent signal sources. Optimally, neurostimulator platforms need to process and synchronized bio signals other than brain activity, derived from sensors embedded in the neurostimulator itself (e.g., gyroscope) or from peripheral sensors. In addition, the patient should be part of the loop, as important feedback on treatment satisfaction could be provided by interactive and patients suitable apps. Finally, the multidimensionality of multi-objective control requires simple and intuitive integrative platforms that can be efficiently handled and adjusted by the medical personnel.

## Multi-Layer Closed-Loop Deep Brain Stimulation: a Precision Medicine Approach

For over 30 years, DBS therapies have been restricted to continuous paradigms. Advances in implantable technology now offer the possibility to monitor and control neural signatures in chronically implanted patients, providing the technical substrate to deploy truly personalized therapies. More than ever, it is important to draw awareness on the multi-faceted and dynamic nature of clinical and neurophysiological manifestations. A conceptual framework is critical to steer the development of therapies that can manage multiple dynamical objectives in parallel and integrate existing closed-loop strategies into a clinically relevant therapeutic portfolio. Modularity will play a key role in rendering these approaches manageable, allowing to easily select and tune therapies that operate on multiple temporal layers, and linking them to patient-specific electro-clinical profiles. While several technological and neurophysiological advances are still needed to enable nested multilayer control capabilities, hardware, software and therapeutic developments will need to go hand in hand. The proposed conceptual framework may thus represent an integral part next generation precision medicine instruments.

## Data Availability Statement

The original contributions presented in the study are included in the article/supplementary material, further inquiries can be directed to the corresponding author/s.

## Author Contributions

GT and EMM contributed to the conceptual design, writing, editing, and generation of figures for the manuscript. All authors contributed to the article and approved the submitted version.

## Conflict of Interest

The authors declare that the research was conducted in the absence of any commercial or financial relationships that could be construed as a potential conflict of interest.

## Publisher’s Note

All claims expressed in this article are solely those of the authors and do not necessarily represent those of their affiliated organizations, or those of the publisher, the editors and the reviewers. Any product that may be evaluated in this article, or claim that may be made by its manufacturer, is not guaranteed or endorsed by the publisher.

## Abbreviations

DBS: deep brain stimulation
PD: Parkinson’s disease
LFP: local field potentials
STN: subthalamic nucleus.
